# Design of electromagnetic metasurface using two dimensional crystal nets

**DOI:** 10.1038/s41598-023-32660-y

**Published:** 2023-05-04

**Authors:** Jie Hou, Xiaohong Zhang, Ying Guo, Rui-Zhi Zhang, Meng Guo

**Affiliations:** 1 Shandong Computer Science Center (National Supercomputer Center in Jinan), Qilu University of Technology (Shandong Academy of Sciences), Jinan, 250101 China; 2 The Research Institute for Special Structures of Aeronautical Composite AVIC, Jinan, China; 3 School of Physics, Northwest University, Xi’an, 710069 China

**Keywords:** Metamaterials, Metal-organic frameworks, Two-dimensional materials

## Abstract

Metasurfaces are of great interest as they exhibit unique electromagnetic properties. Currently, metasurface design focuses on generating new meta-atoms and their combinations. Here a topological database, reticular chemistry structure resource (RCSR), is introduced to bring a new dimension and more possibilities for metasurface design. RCSR has over 200 two-dimensional crystal nets, among which 72 are identified as suitable for metasurface design. Using a simple metallic cross as the metaatom, 72 metasurfaces are constructed from the atom positions and lattice vectors of the crystal nets templates. The transmission curves of all the metasurfaces are calculated using the finite-difference time-domain method. The calculated transmission curves have good diversity, showing that the crystal nets approach is a new engineering dimension for metasurface design. Three clusters are found for the calculated curves using the K-means algorithm and principal component analysis. The structure–property relationship between metasurface topology and transmission curve is investigated, but no simple descriptor has been found, indicating that further work is still needed. The crystal net design approach developed in this work can be extended to three-dimensional design and other types of metamaterials like mechanical materials.

## Introduction

Metasurfaces are two-dimensional metamaterials that are widely used in electromagnetic applications^1,2^. Metamaterials were first proposed and fabricated two decades before^3,4^. Afterwards, they generate general interest as they have properties not found in naturally occurring materials. Metamaterials’ properties are not from the properties of the component materials but from the purposely designed structures, therefore, there is a great interest in designing these metamaterials^5^. Currently, most metamaterials design effort focuses on metaatom design and the combinations of metaatoms^6,7^, and modern optimization method and generative models can now handle complex high-dimensional design space and propose metaatoms with desired properties^8,9^. It will be beneficial to introduce a new degree of freedom for design which will introduce more design space and possibly better properties.

**Figure 1:**
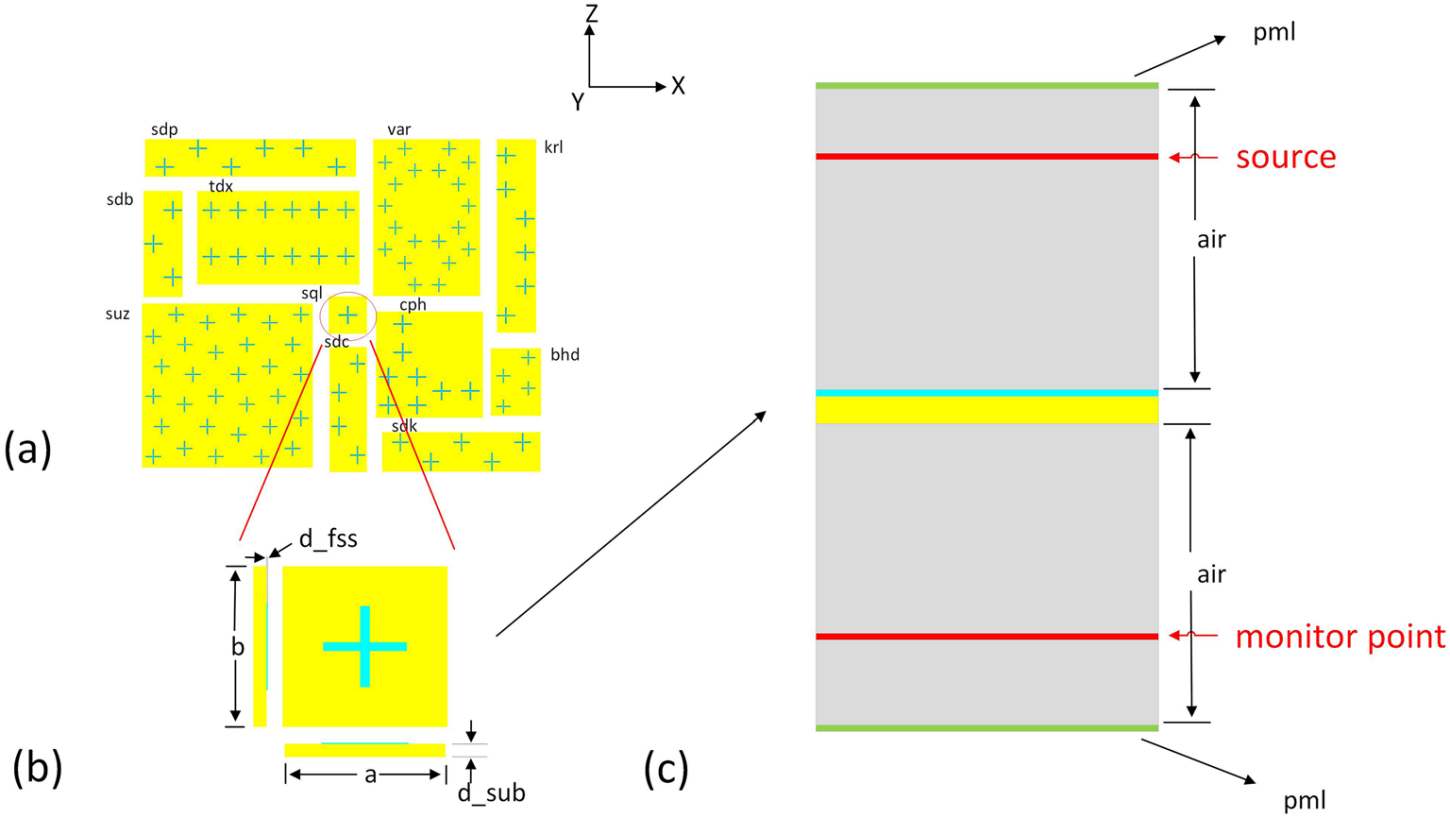
(**a**) Several representative metasurfaces constructed using RCSR crystal nets as templates. Yellow is the glass substrate and cyan crosses are metal. The 3-letter names are crystal net names from RCSR (see text for details). The ratio of metaatoms’ total area to substrate area is set to be the same for all the metasurfaces. (**b**) Cross-sectional unit cell used in MEEP calculations. Crystal net *sql* is shown as an example as it is used in most metasurface. The lattice parameters *a* and *b* are 8 mm for *sql*. The thickness of the metasurface (*d_fss*) is 0.05 mm and the thickness of the substrate (*d_sub*) is 0.4 mm. For all the metasurfaces, *d_fss* and *d_sub* are kept the same. (**c**) The whole air-box along *z*, and the source is a plane wave incident along z. “pml” means perfect match layer. Periodic conditions are applied in x and y directions so the metasurface is 2D infinite. The distances from the metaatom to the source and monitor are 48 mm and 42 mm, respectively. The thickness values of two air boxes and two pml layers are 60 mm and 1 mm, respectively.

The arrangement of metaatoms is a new degree of freedom to explore. It has been shown that when changing the arrangement (i.e. the way that the metaatoms are packed), the transmission curve could be dramatically changed^10^. A recent study designed Moiré metasurface, an idea from twisted bilayer atomic two-dimensional (2D) materials, to provide the surface impedance profiles and generate desired radiation patterns^11^. These inspired us to use crystal nets in reticular chemistry structure resource (RCSR)^12^ for metamaterial design. Crystal nets are topological lattice structures consisting of specific atomic positions and lattice vectors (Fig. 1a). Currently RCSR has more than 8000 three-dimensional (3D) and 200 2D crystal nets, which have been widely used in atomic structure design^13^, especially metal–organic frameworks^14^. To our best knowledge, most metasurfaces use a simple square lattice (RCSR uses “*sql*” to represent *sq*uare *l*attice), and only a few papers consider other types of topological lattice structures for metamaterials design^15,16^. As the 2D atomic lattice structures can be easily transformed into metaatoms and metasurface structures, we expect crystal nets, which describe the topological arrangement of (meta)atoms, to bring a new engineering dimension for metasurface design, like the Moiré metasurface mentioned before.

## Method

### RCSR crystal nets

The 2D crystal nets were downloaded from RCSR website (http://rcsr.anu.edu.au/). Some mistakes were corrected, and the files were converted to JSON format (see “Data Availability” section for the download link). There are 200 two-dimensional crystal nets in the original RCSR dataset, each has a net name, and several representative nets are shown in Fig. 1a. As the RCSR nets have different sizes, the nets in Fig. 1a were purposely selected in order to form a “square shape” for better visualization, and to give an idea that what the nets in RCSR look like. The names of the individual RCSR net are shown next to the structures. Here we briefly described how the crystal nets are named: the 3-letter net names, i.e., RCSR symbols, mostly inherited from zeolite framework type and mineral names, for example, “*dia”* means diamond structure. In some names, there is a hyphen, and the letter after the hyphen describes how the nets are derived from a simple common net (whose name only has three letters), for example *-d* refers to the dual net. More details can be found in the original RCSR paper^12,17^. Each RCSR net has two lattice parameters *a* and *b*, and an example is shown in Fig. 1b.

### RCSR net for metamaterials design

As RCSR is purposely designed for atomic structures, some of the crystal nets might not be suitable to be used as templates for metasurface design. Two criteria were applied to select suitable crystal nets. The first one is based on crystal symmetry. There are five two-dimensional Bravais lattice types (oblique, rectangular, centered rectangular, hexagonal and square). For simplicity with losing generality, only rectangular and square crystal nets are considered. The second criterion is based on vertex distance and metaatom size. In some crystal nets, the vertex is too close (i.e., the distance between two metaatoms is smaller than metaatom size), so the metaatoms centered in these vertices are overlapped. These crystal nets were removed. After applying the two criteria, only 72 crystal nets were kept for further investigation. The crystal nets in Fig. 1a all belong to these 72 selected nets.

To make a direct comparison of metasurfaces using different crystal nets, the lattice parameters of the metasurfaces unit cells are normalized based on metaatom “density” on the substrate. The reference structure is a simple square lattice (shown in net name *sql* in Fig. 1b). The lattice parameters of this structure are set to 8 mm, so the substrate area is 8 × 8 = 64 mm^2^. There is only one metaatom in the metasurface unit cell, so the metaatom “density” is 1 per 64 mm^2^. All other 71 metasurfaces are normalized to make their metaatom density equal to this value. This is done by changing the in-plane lattice parameters *a* and *b* of the unit cell, while keeping the *a*/*b* ratio the same as in the original crystal nets.

### Electromagnetic simulation of RCSR-based metamaterials

Greek crosses were used as metaatoms in the metasurfaces. The length of the Greek cross (4 mm) is the same in all 72 metasurfaces. Cross-type metasurfaces or frequency selective surfaces (FSSs) have been thoroughly investigated for decades^10^, here it is chosen due to its simplicity which facilitates the following analysis. The length of the Greek cross is carefully chosen by considering the second criterion in the RCSR net selection process (detailed in the previous part of “RCSR net for metamaterials design”): if the value of the length is too small, the whole structure cannot function properly as a frequency selective unit; if the value is too large, there will be too much overlap between the neighbor metaatoms and hence there will be only a few RCSR nets could be selected. As the metaatom density is fixed to “1 per 64 mm^2^”, we trialed several values and found that 4 mm makes a reasonable balance between the dilemma described above.

The electromagnetic transmission curves were calculated using MEEP (MIT Electromagnetic Equation Propagation)^18^, an open-source software package for electromagnetics simulation via the finite-difference time-domain (FDTD) method. The simulation unit is shown in Fig. 1c. For simplification without losing generality, only the transverse electric (TE) component of the incident plane wave perpendicular to the metasurface and substrate is considered. The frequency range is set to 10—30 GHz, considering the corresponding wavelength with respect to the unit cell size of the metasurface.

### Data and structure analysis

The calculated transmission curves were analyzed using Scikit-learn. K-means algorithm was performed to cluster the structures using the transmission curves. n_cluster = 3 and default values were used. Principal component analysis (PCA) was used to calculate the coordinates for visualization. Euclidean distance was used in both functions as default.

Structural analysis is conducted using pymatgen package^19^, an open-source Python library for materials analysis. The metasurfaces were first converted to 3-dimensional atomic structure by adding a third dimension, i.e. a vacuum layer, which is suitable for pymatgen analysis (see source code for more details). Space group numbers were obtained using SpacegroupAnalyzer module. Inequivalent atoms were identified using get_symmetry_dataset method in SpacegroupAnalyzer module. Metaatom pair distances were calculated using distance_matrix function in the Structure class. The radial distribution function of the metasurface structure was calculated using RadialDistributionFunction implemented in Matminer^20^.

## Results

**Figure 2:**
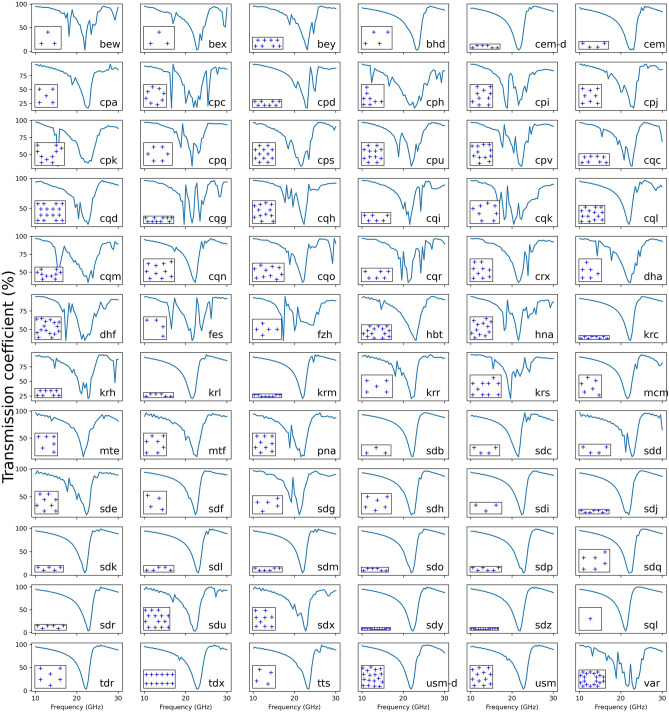
Transmission curves of 72 metasurfaces. The crystal net names from RCSR are shown at the bottom-right corner of each subplot, and the subplots are ordered according to alphabet order of the crystal net names. The crystal net structures are shown at the bottom-left corner of each subplot. It is worth noting that the lattice vectors of the structures is adjusted to fit the figure for best visualization. For a comparison of different structures at the same scale, please see Fig. 1a.

Figure 2 shows the FDTD calculated transmission curves for 72 metasurface structures, and the results are aligned according to the alpha–beta order of crystal net names. The results of *sql* (right-most, second-last row) can be treated as a baseline as the *sql* (square lattice) structure is generally used for most reported metasurface. Figure 2 shows that about one third of the metasurfaces have the same transmission curve as *sql*, for example *bhd*, *cem* and *krl*. The transmission curves in this group share one common feature: there is one and only one valley around 22 GHz.

It is of particular interest how other metasurfaces’ transmission curves are different from that of *sql*. Two groups can be identified: one is represented by *bew*, where the transmission curve has two or more main valleys: beside the valley around 22 GHz (similar to *sql*), there is addition valley around 18 GHz. This feature can also be found in other metasurfaces, to name a few, *cqg, dhf* and *hna*. The other group is represented by *cph*, where the transmission curve has a main valley around 22 GHz and oscillations in other frequencies. To give a quantitative comparison between the curves, clustering algorithms will be used to identify similar curves.

**Figure 3:**
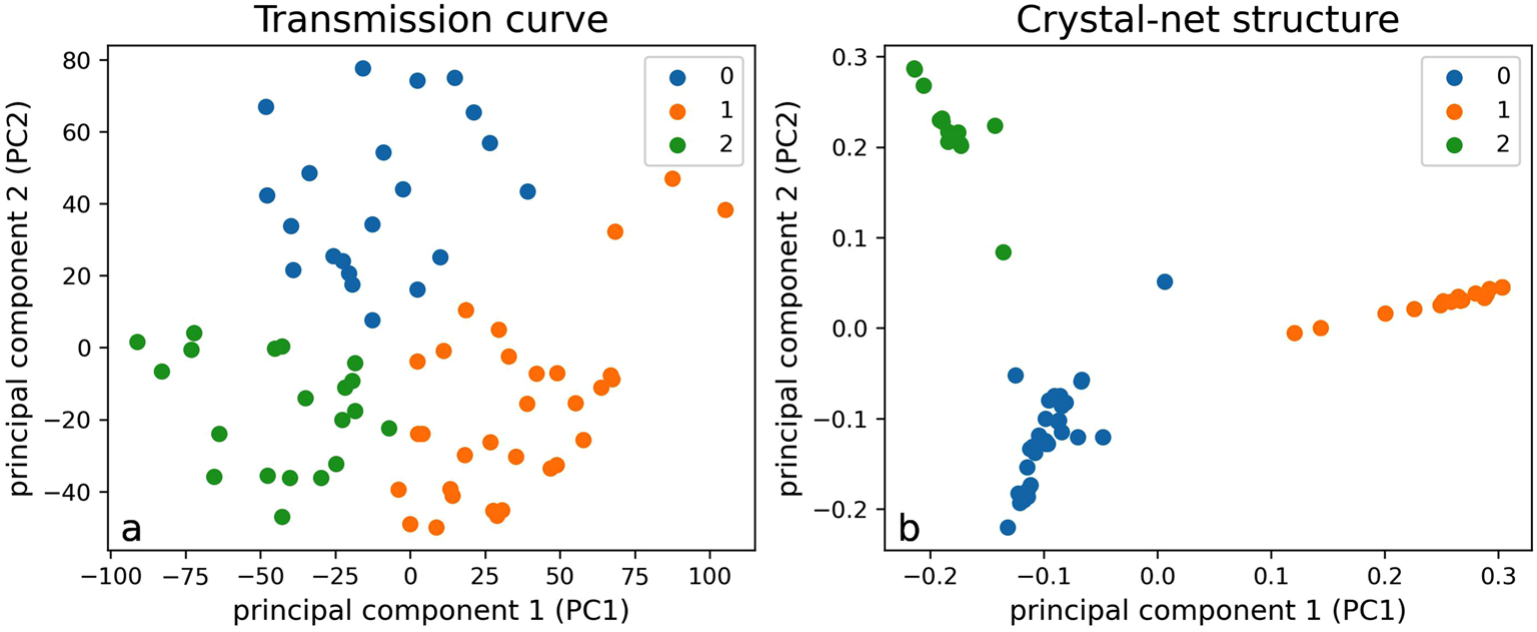
K-means clustering results using (**a**) transmission curve and (**b**) radial distribution function (RDF) of crystal net structures. The number of clusters is set to 3 as it gives the best visualization results, and similar transmission curves (for example “*bew*” and “*cqg*” in Fig. 2) are put into the same cluster and shown in the same color. The coordinates are calculated using principal component analysis (PCA). Principal components 1 and 2 mean the directions along which the data has the most and the second most variation, respectively.

Figure 3a shows the K-means (K is the number of clusters) clustering results of 72 transmission curves. Three K values (2, 3 and 4) were tested, and K = 3 gives the best visualization results, as shown in Fig. 3a. This also agrees with the visual intuition when looking at Fig. 2. The 3 clusters agree with the previous analysis for Fig. 2: the orange, blue or green clusters have transmission curves similar to *sql*, *bew* or *cph*, respectively.

Figure 3b shows clusters of metasurface structures. To calculate the similarity between two metasurfaces, radial distribution function (RDF) is introduced, which is a metric widely used in materials science to measure similarities between different atomic structures^21^. RDF describes how the density of surrounding matter varies as a function of the distance from a point, and it is determined by calculating the distance between all particle pairs and binning them into a histogram. Then the histograms can be fed into K-means and PCA for clustering and visualization using Euclidean distance. As clearly shown in Fig. 3b, the metasurfaces are clustered into three groups using RDFs. Some representative structures in the groups are: *sql* for the blue group, where metaatoms are uniformly distributed in the metasurface; *krm* for the orange group, where metaatoms sit in different rows in a long lattice structure; *cqk* for the green group, where there is a large void inside the lattice. However, the correlations between clustering results of metasurface structures and transmission curves are weak. In other words, the metasurface in the same structural groups does not necessarily have similar transmission curves. Next, we will investigate whether there is some simple relationship between metasurface structures and transmission curves.

**Figure 4:**
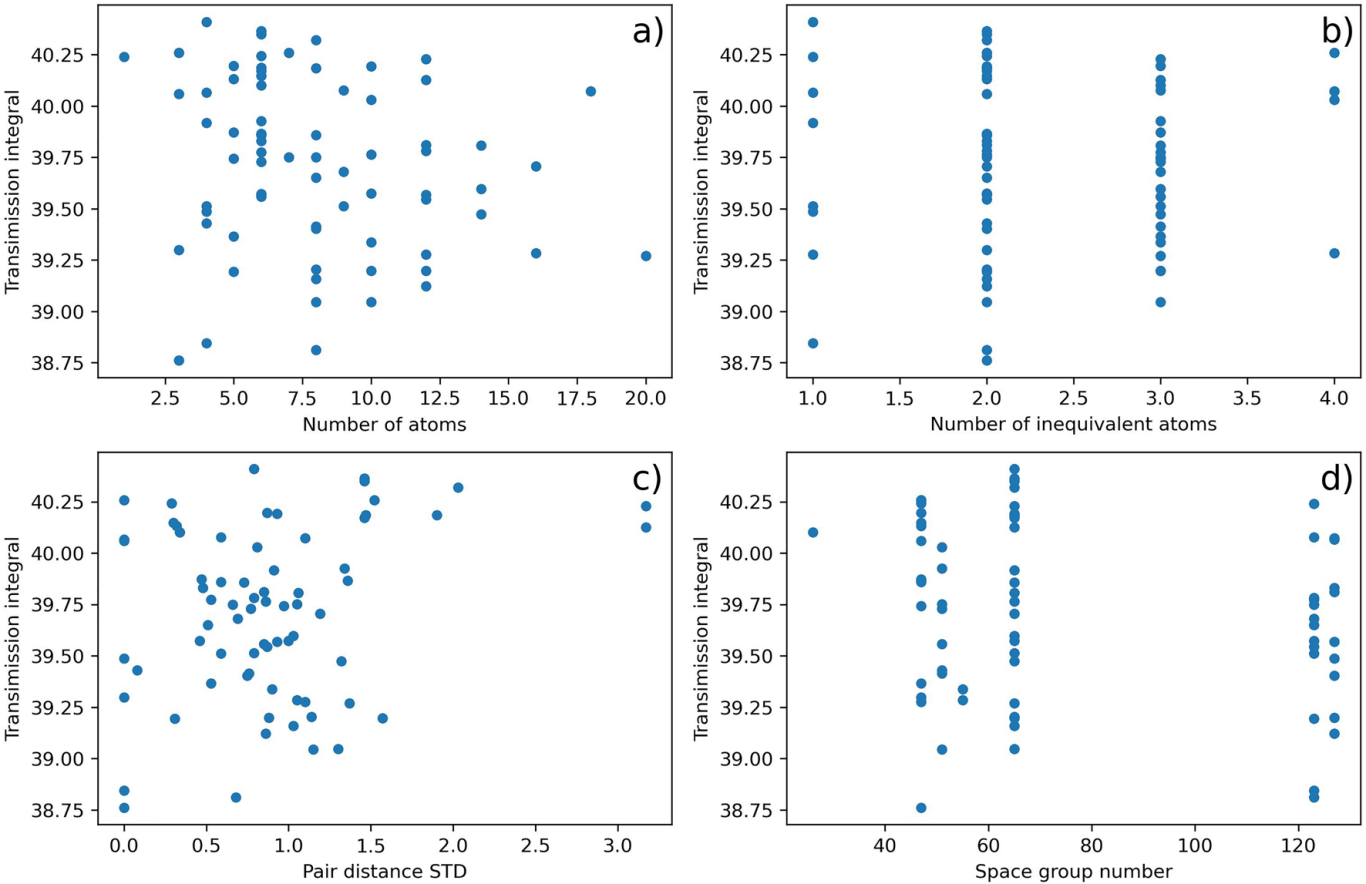
Dependence of transmission curve integral values on crystal net structural features.

To further explore the dependence of transmission curves on crystal net structural features, Fig. 4 plots the integral of the transmission curves (i.e. the area under the curve) against several crystal features typically used in atomic scale materials science. The features are (1) number of atoms: this is the number of crosses in each metasurface, as shown in Fig. 1a and insets of Fig. 2. As the “meta-atom density” are the same for all the metasurfaces (see “Method” Section for details), the number of atoms is also proportional to unit cell size. (2) Number of inequivalent atoms: in one structure, atoms are equivalent if they overlap to each other after a set of symmetrical operations. For example, in “*cpa*” structure, the four atoms at the corners are equivalent to each other, as by rotating 90 degree they overlap to each other. Hence “*cpa*” has 2 inequivalent atoms (while the number of atoms is 5), one in the center and the other at the corner. (3) Pair distance standard deviation: the distance of each pair of cross in one metasurface is calculated, and all the distance values form a “distance matrix” upon which the standard deviation is calculated. This is a measure of the “uniformness” of structure: smaller standard deviation value means the metasurface is more uniform. (4) Space group number: a higher space group number generally means that the metasurface is more symmetric. It is worth noting that 3D space group numbers are used here instead of 2D wall-paper group numbers for simplification, which will not affect the order, i.e., small 3D group number also means small 2D group number.

As shown in Fig. 4, no strong dependence is found for all four structural features. This is not surprising as the “structure–property relationship” between metasurface structure and transmission curves can be complex. Although the four features/descriptors considered here are typical and widely used in materials science^22^, they might not be suitable to describe this relationship, as indicated by Fig. 4. However, it is still possible that suitable descriptor(s) for this relationship can be found, probably by applying feature selection techniques to millions of features. Such an approach has been successful in solid-state materials^23^, while for metamaterial further work is still needed.

## Discussion

Reticular chemistry structure resource (RCSR) is introduced to electromagnetic metasurface design, and transmission curves of 72 metasurfaces constructed using RCSR crystal nets as templates are calculated. The calculated transmission curves are analyzed using K-means clustering and principal component analysis (PCA) for visualization. It is found that the transmission curves can be clustered into three major types. The dependence of transmission curves on metasurface structural features is also investigated. No simple relationship has been found indicating that the structure–property relationship between metasurface and transmission curve can be complex. The calculated transmission curves and the analysis provide basic and necessary information for further applying the topological net to metamaterials design.

The main contribution of this work is providing a systematic way to investigate the new degree of freedom of “lattice structures” for metasurface design. From a “metamaterial data-driven design” point of view, the current work offers a dataset of metasurface structures and calculated transmission curves, which could be a reference for further metamaterial design work using this approach and can be used to build machine learning models. From a practical point of view, the approach introduced in this work provides an alternative way (rather than changing the metaatoms) to modify the transmission curve.

The above point can be further demonstrated by considering an application. In Fig. 2, all the transmission curves have a main valley around 22 GHz, and this feature makes them potential band-stop filters. It is worth noting that the Greek cross is not a state-of-the-art metaatom for band-stop filters, and it was chosen to facilitate high-throughput electromagnetic calculations and to ease the data analysis. Therefore, the purpose here is not to select the best metasurface as a band-stop filter but only to demonstrate the usefulness of the approach. Take *sql* (the square lattice) and *sdq* as examples: compared to *sql*, the metaatoms in *sdq* are shifted, and hence the position of the valley moves right-forward. Another example is comparing more complex patterns of metaatoms arrangement (e.g. *cqm*) to *sql*, the additional valley is introduced. These indicate that RCSR effectively changes the electromagnetic properties of the metasurfaces. Furthermore, if more suitable metaatoms are used, RCSR-based metasurfaces are expected to give better properties for specific applications, for which further work is needed.

Finally, RCSR 2D crystal nets only contain a small fraction of all possible 2D topological lattice structures, and there is a continuous effect to create new 2D nets. Recent progress is the development of a topology-based structure generator^24^ implemented in USPEX package^25^, and this has been used in 2D nanomaterials design^26^. This also offers new opportunities for metasurface and metamaterial design to achieve desired properties.

## Data Availability

The data and python source code generated during the current study are available in the GitHub repository https://github.com/zhangrz1983/RCSR_metasurface.
